# A model for the molecular organisation of the IS*911 *transpososome

**DOI:** 10.1186/1759-8753-1-16

**Published:** 2010-06-16

**Authors:** Philippe Rousseau, Catherine Tardin, Nathalie Tolou, Laurence Salomé, Mick Chandler

**Affiliations:** 1 Université de Toulouse; UPS; Laboratoire de Microbiologie et Génétique Moléculaires, F-31000 Toulouse, France; 2 Centre National de la Recherche Scientifique, LMGM, F-31000 Toulouse, France; 3 Université de Toulouse; UPS; Institut de Pharmacologie et de Biologie Structurale, F-31000 Toulouse, France; 4 Centre National de la Recherche Scientifique, IPBS, F-31000 Toulouse, France

## Abstract

Tight regulation of transposition activity is essential to limit damage transposons may cause by generating potentially lethal DNA rearrangements. Assembly of a bona fide protein-DNA complex, the transpososome, within which transposition is catalysed, is a crucial checkpoint in this regulation. In the case of IS*911*, a member of the large IS3 bacterial insertion sequence family, the transpososome (synaptic complex A; SCA) is composed of the right and left inverted repeated DNA sequences (IRR and IRL) bridged by the transposase, OrfAB (the IS*911*-encoded enzyme that catalyses transposition). To characterise further this important protein-DNA complex *in vitro*, we used different tagged and/or truncated transposase forms and analysed their interaction with IS*911 *ends using gel electrophoresis. Our results allow us to propose a model in which SCA is assembled with a dimeric form of the transposase. Furthermore, we present atomic force microscopy results showing that the terminal inverted repeat sequences are probably assembled in a parallel configuration within the SCA. These results represent the first step in the structural description of the IS*911 *transpososome, and are discussed in comparison with the very few other transpososome examples described in the literature.

## Introduction

Transposons are ubiquitous. They have had and continue to exert a major effect on genome architecture, gene expression and organisation. Tight regulation of their activity is essential to limit damage they may cause by generating potentially lethal DNA rearrangements. Indeed, their study has provided many examples of judicious regulatory mechanisms that are used to achieve this [1].

A crucial checkpoint in transposition is the assembly of the 'transpososome'. This step is a general prerequisite for initiating DNA cleavage and the subsequent chemical steps in transposition, for most elements that use a DNA transposition intermediate. In this protein-DNA complex, both ends of the transposon are bridged by an element-specific enzyme, the transposase, which catalyses the DNA strand cleavages and strand transfers necessary for transposon mobility [2]. The transpososome adopts very precise architectures to accomplish these steps, and undergoes defined changes throughout the transposition process. Such conformational changes have been observed within the transpososome of IS*50 *[3-5], the bacteriophage Mu (which requires three of four specific transposase binding sites) [6] and Tn*10 *(whose transpososome shows an increase in stability as it assembles) [7,8]. Moreover, for both IS*50 *and Mu, a transposase molecule binds to one end but is catalytically active only on the other transposon end. This arrangement ensures that cleavage does not occur [6] before the correct complex has been assembled [9,10]. In spite of its key importance, the composition and organisation of such assemblies have been examined for only a handful of transposable elements [2,11] and with varying degrees of detail.

**Figure 1:**
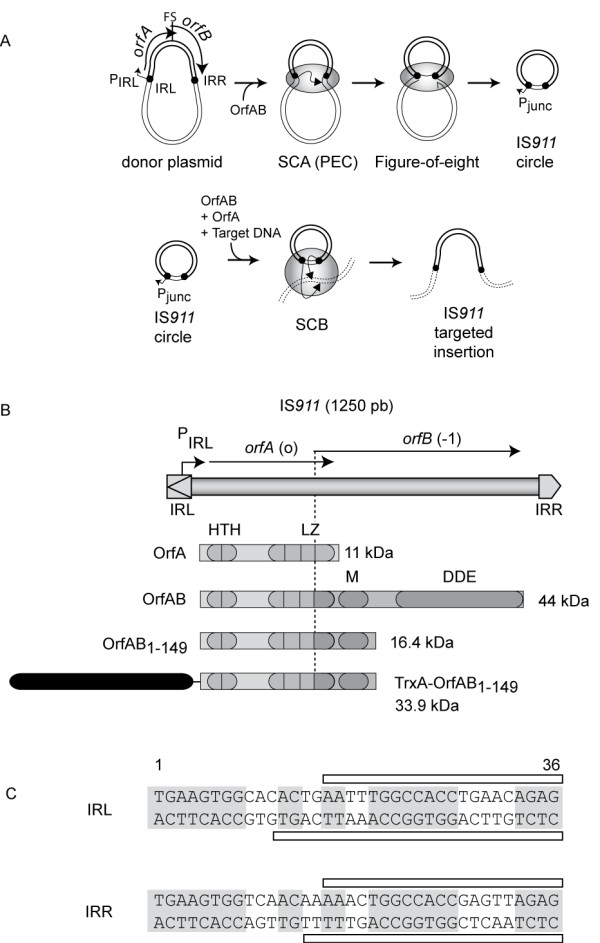
**IS911 genetic organisation transposition pathway**. **(A) **IS*911*, donor DNA and target DNA are represented as bold, fine and dotted lines respectively. Small dark circles represent the two inverted repeats (IRs). Grey ellipses represent OrfAB: note that the stoichiometry is not shown. Arrows represent strand cleavage and transfer reactions that take place within the synaptic complex (SCA) or paired end complex (PEC). IS*911 *insertion is drawn as a nontargeted pathway [16,31,32]. In the synaptic complex B (SCB), proteins are represented as grey ellipses without implying OrfAB stoichiometry or the presence or absence of OrfA in the complex. **(B) **Inverted repeat left and right (IRL, IRR) sequences: *orfA *(translational phase 0) and *orfB *(phase -1). pIRL is indicated, and the frameshift site is shown as a dotted line. HTH motifs are designated as grey ovals and LZ motifs as an ellipse for each heptad. The M and DDE domains are also indicated. Domains present in both OrfA (100 aa) and OrfAB (382 aa) are indicated by light grey, and those in OrfAB alone by dark grey (see text). The two designed protein variant used in this study OrfAB1-149 (149 amino acids (aa)) and TrxA-OrfAB1-149 (308 aa) are represented. The dark box represents the TrxA tag. (c) Nucleotide sequence of the terminal IRL and IRR. Conserved nucleotides are shown on a grey background. The DNaseI footprint of OrfAB[1-149] is indicated schematically above and below the sequences[21].

In this study, we examined the transposition properties of the bacterial insertion sequence, IS*911 *[12]. Bacterial insertion sequences (IS) are among the smallest autonomous transposable elements. IS*911 *belongs to the largest known family of ISs, the IS*3 *family (ISfinder: http://www-is.biotoul.fr). The distinguishing feature of these compared with other well-known ISs is that they undergo transposition through replicative excision and conservative integration (Figure 1a). First, an asymmetric attack of one end by the other leads to the formation of a single-strand bridge between the ends. This resembles a nascent replication fork, which is presumably involved in assembly of a replication apparatus. Replication generates a covalently closed circular copy of the IS in which the left and right ends are abutted and separated by 3-4 bp of DNA originally flanking the IS. The transposon circle subsequently undergoes integration into a target molecule. It is now clear that this mechanism is very widespread and has been adopted not only by members of the IS*3 *family but also by many additional IS families [13-15].

The overall pathway of IS*911 *transposition (Figure 1a) implies the consecutive assembly of two types of transpososome: one ensuring formation of the single-strand bridge (synaptic complex A; SCA) and a second (synaptic complex B; SCB) ensuring integration of the IS into the target DNA. In this paper, we address the stoichiometry of the IS*911 *transposase in the formation of the first transpososome, the SCA. Moreover, we show that a dimer of a truncated form of the transposase (containing the DNA binding but not the catalytic domain), assembles two copies of a DNA fragment including a terminal inverted repeat sequence (IRR) in a parallel orientation.

## Materials and methods

### Bacterial strains, media, plasmids and oligonucleotides

The *Escherichia coli *strains used were JS238, DH5α and Bl21DE3, as previously described [16]. Cultures were grown in Luria broth supplemented, when necessary, with the appropriate antibiotics. Selection was on Luria agar plates supplemented with the appropriate antibiotics: ampicillin (Ap, 100 μg/ml), tetracycline (Tc, 15 μg/ml).

Plasmid pAPT166 was used as a substrate to amplify IRR-100, 150 and 250 DNA fragments [17]. The pET32b::AB149 plasmid was obtained by insertion of an *NcoI-Bam*HI PCR DNA fragment containing the *orfAB149 *(amplified from pLH114 [18]) into the pET32a vector (Novagen). Plasmids pLH114 and pET32b::AB149 were used to prepare OrfAB[1-149] and TrxA-OrfAB[1-149] respectively.

The oligonucleotides used are detailed in Table 1.

**Table 1:** Oligonucleotides used.

Primer	Sequence 5'→3'
IRRa	TGAAGTGGTCAACAAAAACTGGCCACCGAGTTAGAG

IRRb	CTCTAACTCGGTGGCCAGTTTTTGTTGACCACTTCA

O5	GATACTGGAAAAAACTCTAACTCGG

O6	GAATGGACGATATCACTTCCATGACG

O7	TCAACGCATATAGCGCTAGCAGCACG

O200	GAGCCCGTCAGTATCGGCGGAA

### DNA procedures

Standard techniques were used for DNA manipulation and cloning [19]. Restriction and DNA-modifying enzymes were purchased from New England Biolabs (Ipswich, MA, USA). DNA was isolated from agarose gels using a gel extraction kit (QiaQuick; Qiagen, Hilden, Germany) and PCR products were purified using a PCR purification kit (QiaQuick; Qiagen), with plasmid DNA was extracted (Miniprep or Maxiprep kits; Qiagen).

IRR 36 corresponds to the exact sequence of IRR, and was generated by oligonucleotide hybridisation between IRRa and IRRb. IRR 100 was generated by PCR from pAPT166 using the O5 and O6 oligonucleotides to give a DNA fragment composed as follows: 5'-14 bp internal flanking DNA-36 bpIRR-50 bp flanking external DNA. IRR 150 was generated by PCR from pAPT166 using the O5 and O7 oligonucleotides to give the DNA fragment: 5'-14 bp internal flanking DNA-36 bpIRR-100 bp flanking external DNA. IRR 250 was generated by PCR from pAPT166 using the O200 and O6 oligonucleotides to give the following DNA fragment: 5'-164 bp internal flanking DNA-36 bp IRR-50 bp flanking external DNA.

### Purification of OrfAB[1-149] and TrxA-OrfAB[1-149]

OrfAB[1-149] was prepared as described previously [18]. TrxA-OrfAB[1-149] was expressed in a BL21DE3 derivative strain from pET32a::AB149. The purification procedure was the same as for OrfAB[1-149] with the following modifications: lysis was performed in HED0.2K10I buffer (Hepes 250 mM ph7.5, KCl 0.2M, dithiothreitol (DTT) 1mM, EDTA 1 mM, imidazole 10 mM), and DEAE chromatography and ammonium sulfate precipitation steps were replaced by a classical nickel-nitrilotriacetic acid (NiNTa) chromatography method, where the protein is eluted with an imidazole gradient (Qiagen).

### Electromobility shift assay

DNA fragments containing IRR were generated by PCR (IRR-100 [16]) or oligonucleotide hybridisation (IRR-36, IRRa + IRRb heated together at 98°C for 5 minutes and cooled to room temperature overnight) and end-labeled with γ^32^PATP using a classic kinase reaction). For electromobility shift assay (EMSA) [18], 5 nM of the DNA fragments were incubated with OrfAB[1-149] and/or TrxA-OrfAB[1-149] in a final volume of 8 μl. Complexes were separated in a 5% polyacrylamide gel in Tris-glucose-EDTA (TGE) buffer (12 V/cm at 4°C) for 3 hours. The results were quantified using ImageGauge software (Fuji, Tokyo, Japan).

### Atomic force microscopy imaging

The sample (in 14 nM DNA R250, 300 nM orfAB149, 25 mM Hepes pH7.5, 200 mM KCl, 1 mM DTT, 1 mM EDTA, 10% glycerol) was incubated at 37°C for 30 min before being diluted 1:10 in atomic force microscopy (AFM) buffer (5 mM Hepes pH7.5, 10 mM MgCl2) and immediately deposited on freshly cleaved mica that had just been pretreated with 0.1 mg/mL polylysine (Sigma-Aldrich, Missouri, MO, USA). After 30 seconds of incubation, the mica was rinsed with an extensive flow of water and dried under N2. Imaging was performed in air using an atomic force microscope (Bioscope II; Veeco Instruments, Palaiseau, France) operating in tapping mode, with silicon tips (PPP-NCH-50; Nanosensors, Neuchatel, Switzerland). DNA contours were drawn manually and their length measured using in-house software (Labview).

## Results

### OrfAB149 binds a 36 bp IRR DNA fragment

Figure 1b illustrates the organisation of IS*911*, which is bordered by short (36 bp) terminal inverted repeats (IR). The transposase, OrfAB, is produced from two reading frames or*fA *and or*fB *by programmed -1 translational frameshifting. The product of the or*fA *frame, OrA (100 aa), is a regulator of transposition. Production of OrfAB (292 aa) alone can be accomplished by inserting a single base pair into the frameshift region to artificially fuse the A and B frames without any change in the OrfAB amino acid sequence [20]. We used a derivative of OrfAB, OrfAB[1-149], truncated for its catalytic (DDE) domain but including the N-terminal specific DNA binding domain (helix-turn-helix; HTH) and multimerisation domains (leucine zipper (LZ); multimerisation (M) [18]) as shown in Figure 1b. Indeed, in our DNA-binding assay (electrophoretic mobility shift assay; EMSA), the full-length OrfAB transposase does not efficiently form stable and specific DNA-protein complexes whereas the truncated form OrfAB[1-149] does [18,21]. This is presumably due to the fact that the OrfAB DNA binding domain is masked by the natural C-terminal catalytic domain after folding (Duval-Valentin *et al*., in preparation). Previous DNase I protection experiments have demonstrated that the truncated form, OrfAB[1-149], binds the 36 bp IS*911 *terminal IRs [21]. Furthermore EMSA experiments performed with different IRRs containing DNA fragments (100 to 150 bp in size) have shown that OrfAB[1-149] assembles large DNA-protein complexes [22]. Finally, using a mixture of two DNA fragments of different sizes, one of these large DNA-protein complexes was previously shown to include two DNA fragments, and is considered as a valid model for the SCA [22].

**Figure 2:**
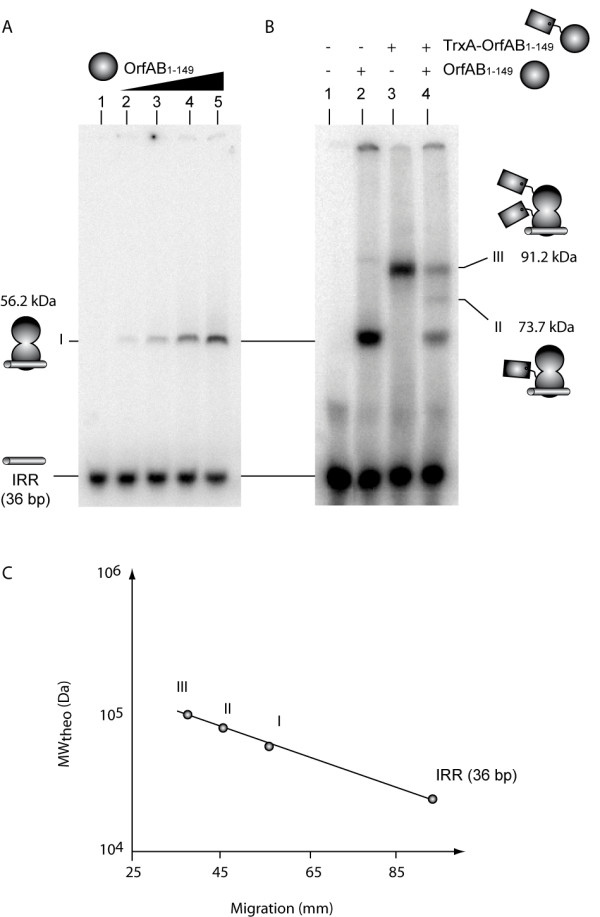
**OrfAB[1-149] binds right inverted repeat (IRR) (36 bp) as a dimer**. **(A) **EMSA analysis of the binding of OrfAB[1-149] (dark circle) to a 36 bp IRR without any flanking DNA (grey cylinder). Radiolabeled IRR containing DNA fragments (5 nM) were incubated with increasing amounts of OrfAB[1-149] (0.06, 0.125, 0.25 and 0.5 μM). Reactions were separated in 5% native polyacrylamide gels (12 V cm) and only one complex (I) is visualised. **(B) **EMSA analysis of the binding of OrfAB[1-149] (dark ball) and TrxA-OrfAB[1-149] (dark ball with a tag) to a 36 bp IRR without any flanking DNA (grey cylinder). Radiolabeled IRR containing DNA fragments (5 nM) were incubated with OrfAB[1-149] and/or TrxA- OrfAB[1-149] (the final protein concentration is 125 nM in lanes 2-4). Reactions were separated on 5% native polyacrylamide gels (12 Vcm-1) and complexes I, II and III are visualised. A model for complexes stoichiometry and molecular weight is proposed. **(C)** The panel represents a plot of the migration (mm) of the complexes in the gel as a function of the logarithm of their theoretical molecular weight as proposed in A.

**Figure 3:**
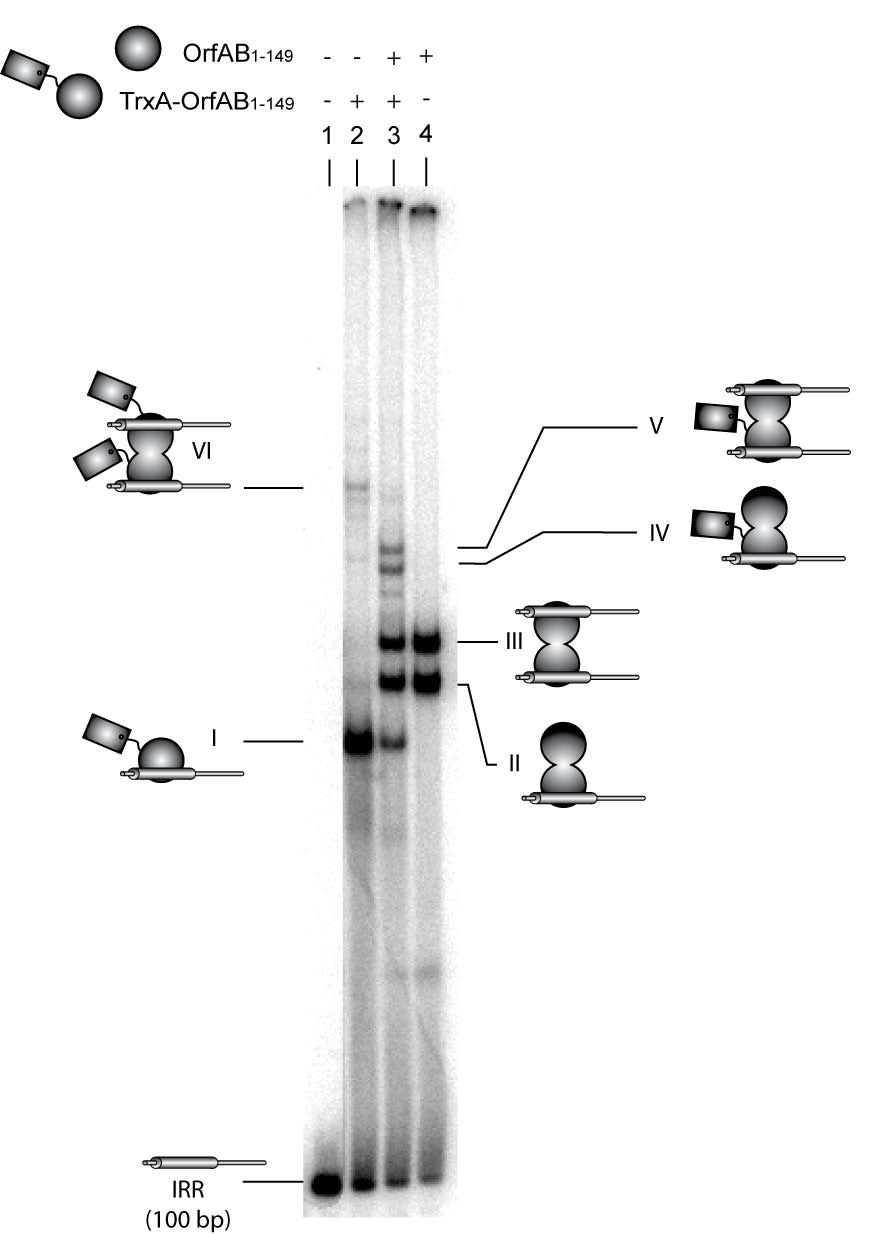
**OrfAB[1-149] synapses right inverted repeat (IRR) (100 bp) as a dimer**. Electromobility shift assay (EMSA) analysis of the binding of OrfAB[1-149] (dark ball) and TrxA- OrfAB[1-149] (dark ball with a tag) to a 100 bp fragment composed of IRR and flanking DNA (large and small grey cylinders respectively). Radiolabeled IRR containing DNA fragments (20 nM) were incubated with OrfAB[1-149] and/or TrxA-OrfAB[1-149] (final protein concentration of 500 nM in lanes 2-4). Reactions were separated in 5% native polyacrylamide gels (12 V/cm), and at least six complexes (from complexes I-VI) visualised. A model for complex stoichiometry is proposed and discussed in the text.

Figure 2a shows the results of an EMSA experiment using a radiolabeled 36 bp oligonucleotide, the precise right end of the IR (IRR), including the entire transposase binding site. We used this minimal fragment to avoid potential additional migratory effects due to the presence of flanking DNA. Only a single complex was observed. The titration experiment (Figure 2a, lanes 2-5) indicated that the truncated OrfAB[1-149] binds less efficiently to this 36 bp IRR fragment than to the longer fragments (≥ 100 bp) [22](see also Figure 3, lane 4). This suggests that flanking DNA sequences included in the longer fragment are important in stabilising OrfAB[1-149] binding [22], probably by providing (nonspecific) interactions. Furthermore, the appearance of only a single retarded species suggests that the DNA fragment containing only IRR, unlike longer DNA fragments ([22] and below), may be too short to form a synaptic complex with two DNA copies. This implies that flanking DNA is not only important for interaction with OrfAB[1-149] but also for allowing synapse formation.

### OrfAB[1-149] binds IRR as a dimer

To determine the stoichiometry of OrfAB[1-149] in this complex, we engineered a derivative including an N-terminal TrxA-tag (see Materials and Methods). The molecular weight of OrfAB[1-149] was thus increased from 16.4 kDa to 33.9 kDa. We used this protein together with OrfAB[1-149] in EMSA experiments (Figure 2b). The effect of increasing the mass of OrfAB[1-149] in the TrxA-OrfAB[1-149] derivative is clearly observable by its enhanced retardation of the protein-DNA complex (compare Figure 2b, lanes 2 and 3). Moreover, when both protein derivatives were included in the reaction mixture, a single complex of intermediate migration could be observed in addition to the high and lower retarded complexes generated, respectively, by TrxA-OrfAB[1-149] and OrfAB[1-149] alone (Figure 2b, lane 4). Note that this result required that the two proteins be pre-incubated together before addition of the DNA. The result is formally consistent with the idea that OrfAB[1-149] binds as a dimer. Moreover when we plotted the logarithm of the predicted molecular mass of the different protein-DNA complexes, based on a 'protein dimer hypothesis', as a function of the migration distance in the electrophoresis gel, we obtained a linear relationship (Figure 3b) [23]. This observation reinforces the idea that the observed DNA-protein complexes are composed of protein dimers.

### OrfAB[1-149] pairs IRR as a dimer

The use of a longer (100 bp) IRR-carrying DNA fragment generated a more complex retardation pattern (Figure 3). Previous results using OrfAB[1-149] together with a longer IR-containing DNA fragment have shown that the SCA can be detected at relatively low protein concentrations, and that increasing OrfAB[1-149] in the reaction mixture generated a complex that migrated slightly faster and that is thought to carry only one DNA fragment (as judged by its behavior when a mixture of fragments of two lengths is used) [22] (Figure 3, lane 4; complexes II and III). The TrxA-OrfAB[1-149] derivative under these conditions generated at least two complexes: relatively high levels of complex I and a very low level of complex VI (Figure 3, lane 2). We propose that complex I is composed of a TrxA-OrfAB[1-149] monomer bound to IRR, whereas complex VI is the synaptic complex. This suggests that TrxA-OrfAB[1-149] binds poorly as a dimer on DNA fragments longer than 36 bp. This may reflect steric hindrance by the Trx tag and flanking DNA. Note that OrfAB[1-149] also forms a small amount of a complex on the 100 bp DNA fragment (located between complex I and free DNA; Figure 3, lanes 3 and 4), which might correspond to a DNA-bound monomer. A mixture of both proteins led to the loss of visible TrxA-OrfAB[1-149] complexes, again reinforcing the idea that TrxA-OrfAB[1-149] alone binds poorly as a dimer on the IRR-containing 100 bp DNA fragment. Two novel complexes were observed (Figure 3, lane 3), consistent with a mixed OrfAB[1-149]/TrxA-OrfAB[1-149] dimer bound to one (complex IV) or two (complex V) DNA fragments. Moreover, the intensity of these bands suggests that TrxA-OrfAB[1-149] binding is enhanced when combined with OrfAB[1-149]. We note that the flanking DNA appears to contribute to the migration properties of the complex in such a way that they no longer migrate as a function of the mass of the complex. This is presumably because the flanking DNA interacts with the protein and changes the overall shape of the complex [24]. We did not attempt to identify the minor bands.

### Parallel alignment of IRR DNA in SCA formed with OrfAB[1-149]

To obtain information on the architecture of the synaptic complex resulting from OrfAB[1-149] binding SCA (Figure 1a), we used AFM to image DNA fragments of 250 bp carrying IRR in the presence or in absence of the protein.

**Figure 4:**
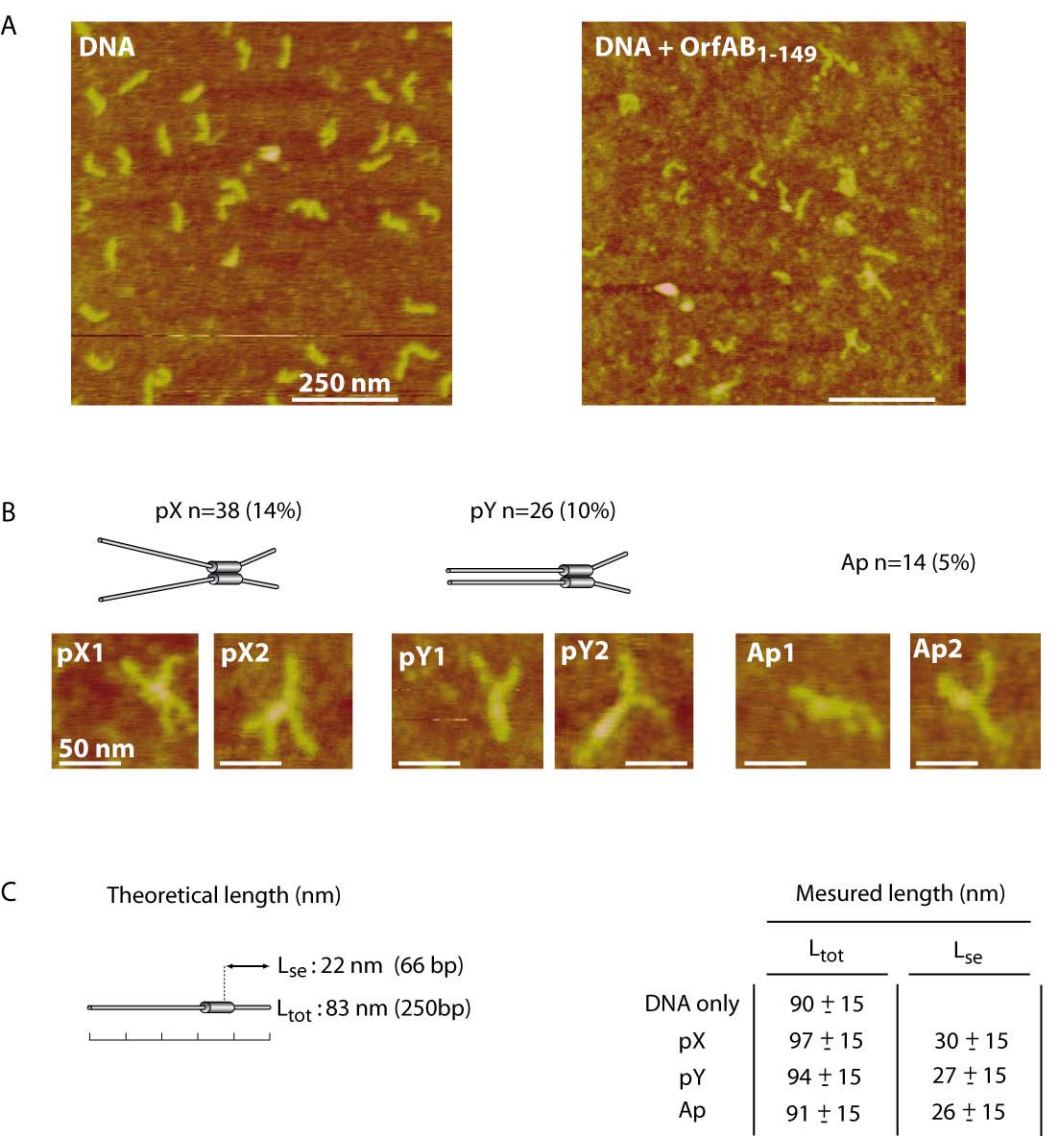
**AFM imaging of IRR-OrfAB[1-149] complexes**. **(A) **Images obtained for a 250 bp DNA fragment carrying a right inverted repeat (IRR), with or without incubation with OrfAB[1-149]. **(B) **Examples of structures observed between OrfAB[1-149] and DNA. DNA is represented as in figures 2-4 as large cylinders (IRR) and small cylinders (flanking DNA). The different structures are: pX = parallel X; pY = parallel Y; Ap = anti-parallel (see text). The Number (n) and percentage of these complexes are given. **(C) **Organisation of the DNA fragment is represented with theoretical lengths: L_tot _is the total length and L_se _is the length from the end of the DNA fragment to the OrfAB[1-149] binding site within IRR (Figure 1c).

In the absence of protein, we observed many DNA molecules spread over the entire field of the mica surface (Figure 4a, left panel). The contour length from one end to the other of the individual molecules was 90 ± 15 nm (n = 283), which is compatible with the theoretical size of the DNA molecules (250 bp = 83nm, Figure 4c, Figure 4d). In fact, it is known that in the conditions used here, AFM imaging of DNA molecules overestimates the real size [25].

When OrfAB[1-149] was added to the DNA solution at the same concentration before deposition on mica (Figure 4a, right panel), many DNA molecules could also be observed (n = 272) but 24% of these were found to cross each other compared with only 6% in the absence of protein. Although the protein was not always clearly visible on these 'crossed' DNA molecules, we believe that they are indeed OrfAB[1-149]-mediated DNA synapses.

The DNA molecules used here were asymmetric with respect to the 20-25 bp OrfAB binding site [21], which was located at a distance of 66 bp (22 nm) from the closest DNA end (Figure 4c). This asymmetric arrangement in principle permits determination of the relative orientation of the two DNA molecules in the synaptic complex. The distance from the intersection point to the shortest end (L_se_) of the crossed molecules was found to be 31 ± 14 nm (n = 78), in good agreement with that expected for OrfAB[1-149]-mediated synapses (Figures 4b, Figure 4c).

A more detailed analysis of the length of the arms in the sample of 78 crossed molecules revealed two morphologies. The majority (n = 64) were composed of DNA molecules aligned in a parallel fashion whereas the other 14 appeared to be organised as antiparallel complexes. Of those aligned in parallel, 38 had an X configuration (Figure 4b, the so-called parallel X (pX)) and 26 had a Y configuration so that the DNA 'tails' formed a single branch (Figure 4b; parallel Y (pY)). The measured L_se _of the pX and pY complexes was 30 ± 15 nm and 27 ± 15 nm, respectively, compatible with complexes in which external flanking DNA is never paired but the internal flanking DNA is paired in the pY population.

Taken together, these results suggest that OrfAB[1-149] promotes the formation of parallel paired DNA molecule complexes, and it seems probable that synaptic complex A of IS*911 *generated by the full-length transposase would also assume this configuration. We believe that the minor fraction of apparently antiparallel structures consists of nonspecific products rather than real synaptic complexes.

## Discussion

The asymmetric two-step transposition mechanism adopted by IS*911 *(Figure 1a) is one of the most extensively used pathways by known ISs. In addition to the IS*3 *family (505 members identified; http://www-is.biotoul.fr) to which IS*911 *belongs, members of the IS*21 *(130 members), IS*30 *(78 members) and IS*256 *(113 members) families also appear to use similar mechanisms [12-15]. This contrasts with the other transposition paradigms of both prokaryotic (IS*10*, IS*50*, Tn*7*) and eukaryotic (mariner, hermes, PiggyBac) origin, which shed their flanking DNA without forming a covalently circular intermediate, and of bacteriophage Mu and members of the Tn3 transposon family, which retain their DNA flanks during transposition to generate co-integrated intermediates with fused donor and target molecules [26-28].

The IS*911 *model therefore represents an important and widespread transposition paradigm. Because the IS*911 *transposase (OrfAB) like most transposases of this type, has to date proved refractory to structural analysis by classic X-ray crystallography and nuclear magnetic resonance approaches, we adopted alternative methods here to probe the organisation of the IS*911 *transpososome. We used a minimal DNA fragment of 36 bp constituting the right terminal IS*911 *inverted repeat, IRR and analysed the formation of protein-DNA complexes (Figure 2). With this short DNA, OrfAB[1-149], a C-terminal truncated OrfAB derivative carrying the first 149 amino acids (Figure 1b), appeared unable to form synaptic complex A, in which the two ends are bridged by the truncated transposase derivative. Using larger DNA fragments (carrying flanking DNA on each end of IRR), SCA were readily observed with OrfAB[1-149] [21]. Gel retardation with OrfAB[1-149] and an OrfAB[1-149] derivative with an N-terminal TrxA extension indicated that OrfAB[1-149] was present as a dimer. A mixture of both OrfAB[1-149] and TrxA-OrfAB[1-149] generated a single additional band compared with those generated by the two proteins separately (Figure 2). These experiments also revealed that the 36 bp DNA fragment showed poor affinity for the proteins and suggested that the TrxA-OrfAB[1-149] derivative bound even less avidly, presumably as a result of steric hindrance due to the presence of the TrxA tag.

Importantly, the degree of migration of the three complexes was consistent with their proposed composition: complex I, the OrfAB[1-149] dimer; complex II, the OrfAB[1-149]+TrxA-OrfAB[1-149], and complex III, the TrxA-OrfAB[1-149] dimer. Results using larger DNA fragments (Figure 3) suggested that SCA also includes two OrfAB[1-149] molecules, although the migration of the complexes could not be used to determine their molecular mass. This is presumably due to the presence of additional flanking DNA that acts to increase the stability of the protein-DNA complex. The additional protein-DNA interactions that may occur to achieve this may change DNA configuration (for example, by bending or wrapping the DNA round the protein) and therefore exert a major influence on complex migration through the gel [8,24].

Having established that two OrfAB[1-149] monomers bridge the two IRR-containing DNA fragments, we initiated AFM studies in an attempt to determine the configuration of DNA molecules in these complexes. We were able to demonstrate that the addition of OrfAB[1-149] to a 250 bp DNA carrying an IRR resulted in a very significant increase in the percentage of paired DNA (24% versus 6%). Moreover, because the position of the OrfAB[1-149] binding site was asymmetric, we were able to assess whether the DNA in these complexes was in a parallel or an antiparallel configuration. As judged by this two-dimensional imaging technique, the vast majority of these potential synaptic complexes appeared to contain DNA in a parallel configuration. They showed two types of morphology: either Y- (pY) or X-like (pX). In both cases, the position corresponding to the OrfAB[1-149] binding site within IRR was paired whereas the external flanking DNA was always unpaired. The difference between pX and pY depends on whether the DNA portion corresponding to the internal flanking DNA is paired or not; this DNA is part of IS*911 *but not part of IRR. Our failure to clearly observe OrfAB[1-149] by AFM might be due to its small size (17.5 kDa) and the involvement of only one protein dimer per SCA as shown above. It could also stem from a partial disruption of the proteins from the DNA during dilution and deposition of the complexes, even though the polylysine-treated mica support is expected to trap the DNA instantly [29].

This result is consistent with the observation obtained from measurement of the relative efficiencies of synaptic complex formation/stability between inverted and directly repeated pairs of IS*911 *ends. Using tethered particle motion to measure the length of a tethered DNA molecule by the trace of a bead attached to its free end, we showed that although OrfAB[1-149] can bridge two directly repeated ends (measured by the shortening due to the resulting DNA loop), it does so with less efficiency than for a DNA substrate carrying inverted ends [30]. The observed dynamics of SCA formation were more compatible with parallel rather than antiparallel pairing of IRR and IRL in the SCA. Both this and the result obtained using AFM suggest that the ends are brought together in a parallel configuration.

These observations on SCA organisation are in agreement with previously proposed models based on EMSA, DNAse protection and deletion experiments [21]. We propose that a dimer of OrfAB[1-149] binds and bridges a subterminal region within inverted repeats (IRR and IRL) to form the SCA. This explains the functional importance of the LZ and M multimerisation domains of OrfAB[1-149], which cannot be mutated or deleted without destroying specific DNA binding activity of the protein [22]. The fact that the external region of IRR and the external flanking DNA were never observed to be bridged in the AFM images and are not protected against DNase I in footprinting experiments [21] implies that the terminal regions of the IR are free for contacts with the catalytic domain of OrfAB, which is absent in OrfAB[1-149]. This type of configuration would also permit *trans *cleavage, as observed for IS*50 *[9] and bacteriophage Mu [10], in which the catalytic domain of one transposase molecule bound at one end is directed to cleave the opposite IS end. Experiments are in progress to test these predictions.

## Competing interests

The authors declare that they have no competing interests.

## Authors' contributions

PR, CT and NT carried out the experiments. PR, CT and MC conceived the experiments. PR, MC and CT wrote the manuscript. MC and LS provided funding and facilities.
